# Human ILC3 Exert TRAIL-Mediated Cytotoxicity Towards Cancer Cells

**DOI:** 10.3389/fimmu.2022.742571

**Published:** 2022-03-01

**Authors:** Jana-Julia Siegler, Margareta P. Correia, Tomáš Hofman, Isabel Prager, Emrullah Birgin, Nuh N. Rahbari, Carsten Watzl, Ana Stojanovic, Adelheid Cerwenka

**Affiliations:** ^1^ Department of Immunobiochemistry, Medical Faculty Mannheim, University of Heidelberg, Mannheim, Germany; ^2^ Mannheim Institute for Innate Immunoscience (MI3), Medical Faculty Mannheim, University of Heidelberg, Mannheim, Germany; ^3^ Cancer Biology and Epigenetics Group, Research Center of IPO Porto (CI-IPOP)/RISE@CI-IPOP (Health Research Network), Portuguese Oncology Institute of Porto (IPO Porto)/Porto Comprehensive Cancer Center (Porto.CCC), Porto, Portugal; ^4^ Department for Immunology, Leibniz Research Centre for Working Environment and Human Factors at TU Dortmund (IfADo), Dortmund, Germany; ^5^ Department of Surgery, University Clinics Mannheim, Medical Faculty Mannheim, Heidelberg University, Mannheim, Germany; ^6^ European Center for Angioscience (ECAS), Medical Faculty Mannheim, University of Heidelberg, Mannheim, Germany

**Keywords:** NK cells, ILC, ILC3, TRAIL, cytotoxicity, innate immunity

## Abstract

Group 3 helper Innate Lymphoid Cells (ILC3s) are cytokine-producing lymphocytes that respond to stress signals released during disturbed tissue homeostasis and infection. Upon activation, ILC3s secrete IL-22 and IL-17, and orchestrate immune responses against extracellular pathogens. Their role in cancer remains poorly explored. To determine their anti-cancer effector potential, we co-cultured cytokine-activated human ILC3s with cancer cells of different origins. ILC3s were able to directly respond to tumor cells, resulting in enhanced IFN-γ production. Upon tumor cell encounter, ILC3s maintained expression of the transcription factor RORγt, indicating that ILC3s preserved their identity. ILC3s were able to directly kill both hepatocellular carcinoma and melanoma tumor cells expressing cell-death receptor TRAILR2, through the activation of Caspase-8 in target cells. Moreover, liver-derived cytokine-activated ILC3s also expressed TRAIL and were able to eliminate hepatoblastoma cells. Together, our data reveal that ILC3s can participate in anti-tumor immune response through direct recognition of tumor cells resulting in IFN-γ release and TRAIL-dependent cytotoxicity. Thus, ILC3s might be ancillary players of anti-tumor immunity in tissues, acting as primary responders against transformed or metastasizing cells, which might be further exploited for therapies against cancer.

## Introduction

The family of innate lymphoid cells (ILCs) is a heterogeneous group of immune cells belonging to the innate immune system. They lack the somatic rearrangements of the antigen receptor genes, as described for B or T cells, and comprise cytotoxic natural killer (NK) cells, lymphoid tissue inducer cells (LTis), and several subsets of helper innate lymphocytes (1). LTis and helper ILCs express CD127 (IL-7Rα) and, unlike NK cells, depend on the cytokine IL-7 for their development (1, 2). Helper ILCs are further subdivided based on transcription factor expression and their cytokine production profiles. Group 1 ILCs (ILC1s) express T-bet and produce TNF-α and IFN-γ (3, 4), while group 2 ILCs (ILC2s) express GATA3 and produce IL-4, IL-5 and IL-13 (5–7). Group 3 ILCs (ILC3s) express RORγt, which is required for their development and function (8, 9), and, upon stimulation with IL-1β and IL-23, produce IL-22 and IL-17. Additionally, ILC3s were reported to secrete GM-CSF, TNF-α, IL-8 and IFN-γ (10–12).

ILC3s support tissue repair and immunity against extracellular pathogens (13). However, their role in the tumor immunity is incompletely understood. Several studies reported ILC3 infiltration into the tumor tissue (14–16), but data so far indicate an ambiguous role of ILC3s in cancer (17). It has been shown that ILC3s can both promote and inhibit tumor growth (18, 19). In particular, they were reported to promote the development of colon cancer (19) and the proliferation of pancreatic cancer cells (16) *via* the production of IL-22. In the context of liver cancer, it was shown that IL-23-stimulated mouse ILC3s promoted the development of hepatocellular carcinoma (HCC) *via* IL-17 production (20). In addition, ILC3s were described to facilitate the formation of lymph node metastasis of breast cancer *via* the interaction with stromal cells (21). On the other hand, NKp46+ ILC3s were crucial for IL-12-dependent tumor suppression in a mouse model of melanoma (18). Recently, it was shown that tonsillar CD94+ ILCs could kill human leukemia cells, and IL-12 could induce this phenotype in non-cytotoxic fraction by upregulating cytolytic machinery and several NK cell-expressed molecules, including CD94 and Eomes (22). Moreover, human NKp44+ ILC3s were shown to support the formation of protective tertiary lymphoid structures and thereby constrain tumor growth of lung (14) and colorectal cancer (23).

Here, we demonstrate a direct cytotoxic effector potential of human ILC3s towards tumor cells. Our data reveal that blood-derived human ILC3s can directly respond to tumor cells by increased production of IFN-γ, and cytotoxicity mediated *via* the interaction of TRAIL and its receptor on cancer cells. We show that both intra-hepatic and blood-derived cytokine-stimulated ILC3s are able to kill hepatic tumor cells, which holds promise for the exploitation of the cytolytic and cytokine-producing potential of ILC3s as additional immune effectors against cancer.

## Material And Methods

### Cell Lines

The human hepatocellular carcinoma cell line (HepG2) and the hepatoblastoma cell line (HuH6) were cultured in DMEM medium (Sigma-Aldrich) supplemented with 10% FCS, 1% penicillin/streptomycin and 1X non-essential amino acids (all from Gibco). The human melanoma (SK-Mel-37 and SK-Mel-28.B7H6) and cervical carcinoma (HeLa-CD48) (24) cell lines were cultured in RPMI 1640 medium (Sigma-Aldrich) supplemented with 10% FCS and 1% penicillin/streptomycin. Cells were dissociated with trypsin or with non-enzymatic dissociation solution (both from Sigma-Aldrich).

### Generation of CRISPR/Cas9-Mediated TRAILR2 Knockout Cell Lines

TRAILR2-deficient cancer cell lines were generated as described (25). HepG2, HuH6 and SK-Mel-37 cells were first transduced with the lentiCas9-Blast (Addgene #52962) vector, encoding Cas9 protein and Blasticidin-resistance gene, used to select Cas9-expressing cells. For generating the TRAILR2 knockout cell lines, sgRNA were designed using the https://design.synthego.com/#/ website. Three sgRNA with the lowest off-target ratio were selected and cloned individually into pLenti_HDCRISPR_NoC sgRNA-puromycin expression vector (kindly provided by Prof. Dr. Michael Boutros, DKFZ, Heidelberg). SgRNA-containing vectors were transfected separately into Cas9-expressing cell lines using jetOPTIMUS (Polyplus #117-15). After selection with 2.5 µg/ml of Puromycin (Sigma-Aldrich) for 2 days, the expression of TRAILR2 was measured using flow cytometry, and TRAILR2-negative population was purified by flow cytometric sort from the cell lines with the highest knockout efficiency.

### ILC3 Isolation and Culture

Buffy coats from healthy donors were obtained from DRK-Blutspendedienst Baden-Württemberg-Hessen (Mannheim, Germany). Written informed consent from the blood donors was obtained and ethical approval 87/04 was granted by the Ethik Kommission II of the Medical Faculty Mannheim (Mannheim, Germany). Adjacent non-cancerous tissue from human livers was acquired from patients undergoing surgical resection for hepatocellular carcinoma (HCC) or colorectal cancer liver metastasis. The collection and use of patient material was approved (2012-293N-MA) by the Ethik Kommission II of the Medical Faculty Mannheim. The samples were collected according to the principles of the Declaration of Helsinki. All patients gave their written consent.

Liver samples were cut into small pieces and were enzymatically digested using a gentleMACS™ Octo Dissociator (Miltenyi) to obtain cell suspension. Mononuclear cells from liver and blood were enriched using a gradient centrifugation, and red blood cells lysis. To purify ILCs, non-ILCs (CD3+, CD19+, CD14+ and CD16+ cells) were depleted using magnetic beads (Miltenyi). Blood and liver ILC3s and NK cells were then isolated from ILC-enriched and ILC-depleted fraction, respectively, using BD FACSAria™ Fusion cell sorter.

Freshly isolated ILC3s and NK cells were cultured overnight in RPMI 1640 AQmedia™ (Sigma-Aldrich) supplemented with 10% human serum (PAA) and 1% penicillin/streptomycin. ILC3 cultures were supplemented with 100 ng/ml IL-1β (Miltenyi), 100 ng/ml IL-23 (Miltenyi) and 10 U/ml IL-2 (NIH), while NK cells were maintained in presence of ILC3 supplements (100 ng/ml IL-1β, 100 ng/ml IL-23 and 10 U/ml IL-2) or with 400 U/ml IL-2.

### Co-Culture of ILCs or NK Cells With Tumor Cell Lines and Functional Assays

ILC3s and NK cells were washed and seeded at the indicated ratios with target cells into 96-well v-bottomed plates (Sarstedt) for 24 h in media containing 10 U/ml IL-2 (NIH). The supernatants were collected for further analysis, while the cells were harvested and used for flow cytometry.

To detect cytokines, collected cell-free supernatants were stored at -20°C until further analysis. IFN-γ cytokine concentrations were measured using ELISA (Biolegend) according to manufacturer’s instructions. Measurements of cytokines IL-22, IL-8, GM-CSF, and TNF-α were performed using a bead-based assay (Legendplex, Biolegend) according to the manufacturer’s instructions.

To determine target cell killing, LDH-Glo™ Cytotoxicity Assay Kit (Promega) was used according to the manufacturer’s instructions. ILC3s and NK cells were seeded either alone (control) or together with 1000 target cells in 96-well v-bottomed plates, in a total volume of 100 µl of RPMI 1640 AQmedia™ supplemented with 10% human serum, 1% penicillin/streptomycin and 10 U/ml IL-2. The conditions were run in duplicates. The purity of ILC3s and NK cells (95-98%) was confirmed by flow cytometry. Spontaneous release of the lactate dehydrogenase (LDH) was measured from cultures of target cells without effectors. In order to determine the maximum release of LDH, target cells were lysed in 0.2 % Triton X-100 (Sigma-Fluka) for 15 min at RT. All controls (including medium) were set up in quadruplicates. Luminescence was measured using a plate reader (Tecan). For every time point, LDH activity measured from medium was subtracted from values of all other conditions, and LDH activity in supernatant of effector cells cultured alone was subtracted from the co-culture-derived values. Finally, % specific cytotoxicity was calculated as 100 x [(experimental LDH – minimum LDH)/(maximum LDH-minimum LDH)].

### Flow Cytometry Analysis and Cell Sorting

For cell surface staining, cells were harvested and washed with PBS. 7AAD or Zombie Aqua™ (both from Biolegend) were used for dead cell discrimination, according to manufacturer’s instructions. Samples were stained with αFasL (NOK-1), αGranzyme B (QA16A02), αPerforin (B-D4), αTRAIL (RIK-2), αTRAILR1 (DJR1), and αTRAILR2 (DJR2-4), all purchased from Biolegend. For intracellular staining, cells were fixed and permeabilized with Foxp3/Transcription Factor Staining Kit (eBioscience™) followed by FACS™ Permeabilizing Solution 2 (BD Biosciences). Cells were washed and then incubated with 10% rat serum followed by αRORγt mAb (AFKJS-9, eBioscience) for 20 min at 4°C.

For ILC3 and NK cell sorting, the following antibodies were used: αCD1a (HI149), αCD3 (OKT3), αCD14 (HCD14), αCD19 (HIB19), αCD34 (561), αCD94 (DX22), αCD123 (6H6), αCD45 (HI30), αFcϵR1α (AER-37), αTCRαβ (IP26), αTCRγδ (B1), αCD117 (104D2), αCD127 (A019D5), and αCD56 (MEM-188), all from Biolegend, and αCRTH2 (BM16) from BD Bioscience. When NK cells and ILC3s were analyzed from the same donor, CD94 and CD16 were excluded from the pre-enrichment and Lineage staining antibody cocktail, and the corresponding antibodies were used for flow-cytometric gating: αCD16 (B73.1) and αCD94 (HP-3D9). Data were analyzed using FlowJo™ version 10 (Becton, Dickinson and Company; 2019).

### Time-Lapse Live-Cell Imaging

Time-lapse live-cell imaging analysis was performed as recently published (24). HeLa-CD48 cells, which express NES-ELQTD-GFP-T2A-NES-VGPD-mCherry and CD48, a ligand for 2B4, were seeded into a silicon-glass microchip (size of wells 350 µm). We have confirmed that effector cells (cytokine-stimulated ILC3s) did not express the 2B4 receptor (**Supplementary Figure 1A**). Tumor cells were left to adhere overnight and then washed once with medium. The microchip was placed into an incubation chamber (37°C, 5% CO_2_ and humidity device S1) at the microscope. 20000 ILC3s resuspended in RPMI 1640 AQmedia™ containing 10% human serum, 1% penicillin/streptomycin and 10 U/ml IL-2, were added into the microchip (2-10 ILC3s per micro-well). Imaging was performed using ApoTome System with Axio Observer 7 microscope (Zeiss) with a 20x/0.8 Plan-Apochromat objective, equipped with an incubator chamber and environmental control system (37°C), in a humidified atmosphere with 5% CO_2_. Fluorescence of GFP and mCherry was acquired in line sequential mode. To visualize mCherry and GFP, we used the Colibri7 LED-module 561 (filter set 64 HE LED) and the LED-module 488 (filter set 38 HE LED), respectively. Brightfield was acquired with the TL LED module. Images were captured at a rate of three z-stack every 3 min and z-slices were spaced by 1 µm. Images were acquired with the camera Axiocam 560 mono. All confocal images were analyzed using the ImageJ software (Rasband, W.S., ImageJ, U. S. National Institutes of Health, Bethesda, Maryland, USA, https://imagej.nih.gov/ij/, 1997-2018), as previously described (24, 26). Briefly, reporter cleavage was quantified based on the redistribution of the fluorescence signal from cytosol to nucleus in image stacks from different time points. The fluorescence signal intensity (I) was measured in the cell nucleus and normalized to the cytosolic signal over time (t). The background was subtracted.


$$I_{normalized\;nucleus}=\frac{I_{nucleus}(t)}{I_{cytosol}(t)}$$

### Statistical Analysis

Data were tested for normal distribution using Shapiro-Wilk test. Statistical differences were then evaluated using the appropriate statistical test indicated in each Figure legend. Data are corrected for multiple comparison testing when necessary. Compared experimental groups were considered to be significantly different when p<0.05.

## Results

### ILC3s Increase IFN-γ Release in Response to Tumor Cells

**Figure 1:**
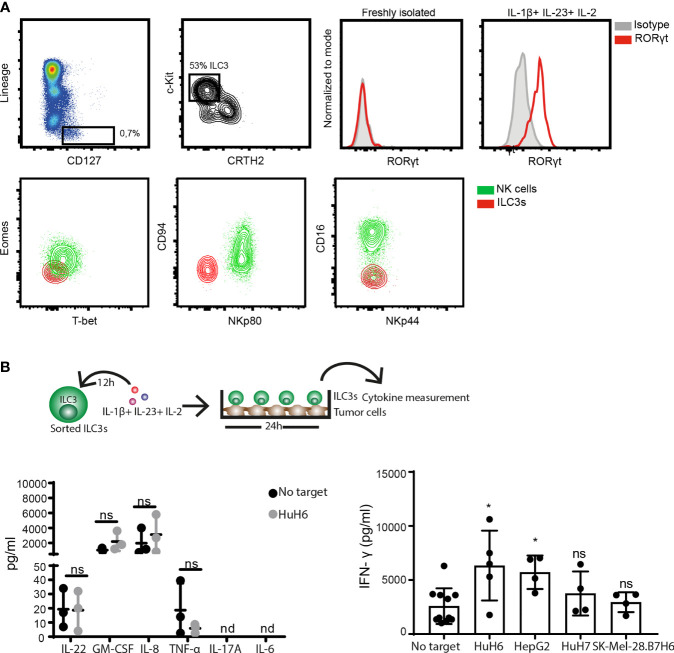
ILC3s increase IFN-γ release in response to tumor cells. **(A)** Representative analysis of blood ILC3s gated as live, CD45^+^, Lineage^-^ (CD3^-^, CD19^-^, CD14^-^, CD34^-^, CD94^-^, CD123^-^, TCR α/β^-^, TCR γ/δ^-^, FCϵR1α^-^), CD127^+^, c-Kit^+^ and CRTH2^-^ cells (left). Histograms show RORγt expression by blood-derived freshly-isolated and ILC3s cultured overnight with 100 ng/ml IL-1β, 100 ng/ml IL-23 and 10 U/ml IL-2 (right). Contour-plots (down) depict expression of Eomes, T-bet, CD94, NKp80, CD16 and NKp44 in gated ILC3s and NK cells (Lin^-^CD127^-^CD56^+^NKp80^+^) from peripheral blood. (**B**, left) Cytokine-treated ILC3s were washed and co-cultured 24h with HuH6 cells in the presence of 10 U/ml IL-2. Concentrations of IL-22, GM-CSF, IL-8, TNF-α were determined in supernatants (n=3 blood donors), ns, not significant, by two-way Anova. (**B**, right) Concentration of IFN-γ was measured in the supernatant of blood ILC3s co-cultured with HuH6, HepG2, HuH7 or SK-Mel-28.B7H6 cells (n=4-5 blood donors), *p <0.05; ns, not significant, by one-way Anova.

To address the ILC3-cancer cell interaction, we first established a co-culture system using ILC3s derived from peripheral blood of healthy donors and hepatic cancer cell lines. In concordance with the literature (27, 28), freshly-isolated circulating ILC3s, gated as viable, CD45+, CD127+, lineage negative (CD3-, CD19-, CD14-, CD34-, CD94-, CD123-, TCR α/β-, TCR γ/δ-, FCϵR1α-) cells, that express c-Kit, but not the ILC2 marker CRTH2, did not express RORγt (**Figure 1A**). Neither expression of the transcription factor Eomes, nor the surface receptors NKp80, CD94, CD16 and NKp44 were detected, while cells expressed T-bet (**Figure 1A** and **Supplementary Figure 1B**). These cells were previously reported to display a multipotent phenotype with the ability to differentiate into all ILC lineages (27). Here, we show that short exposure (16-24h) to type 3-stimulating cytokines IL-1β, IL-23, and IL-2 increased both T-bet and RORγt expression (**Figure 1A** and **Supplementary Figure 1B**). These RORγt-expressing ILC3s produced GM-CSF, IL-8 and IFN-γ, as well as lower amounts of IL-22 and TNF-α, while IL-17 and IL-6 were not detectable (**Figure 1B**). Co-culture with the hepatoblastoma or hepatocellular carcinoma cell lines, HuH6 and HepG2, respectively, increased IFN-γ release by ILC3s, without affecting the production of other cytokines (**Figure 1B**). Thus, ILC3s have the ability to directly recognize and respond to tumor cells, leading to increased IFN-γ production.

### ILC3s Exert Cytotoxicity Against Tumor Cells

**Figure 2:**
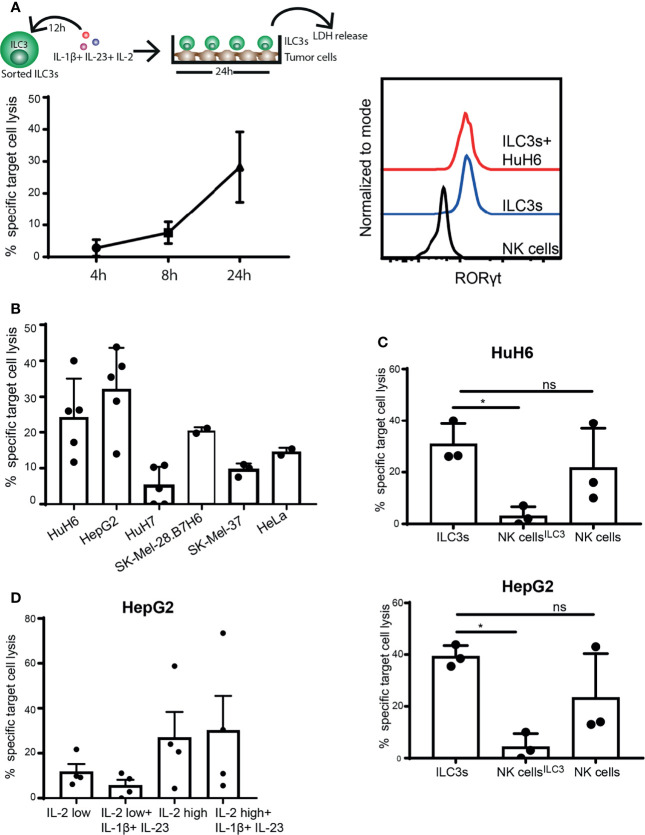
ILC3s exert cytotoxicity against tumor cells. **(A–C)** Sorted blood ILC3s were stimulated overnight with 100 ng/ml IL-1β, 100 ng/ml IL-23 and 10 U/ml IL-2. Afterwards, cells were washed and co-cultured with different tumor cell lines for 24h in the presence of 10 U/ml IL-2. **(A)** Specific target cell lysis of the hepatoblastoma cell line HuH6 after 4h, 8h and 24h of co-culture with ILC3s (n=3 blood donors, E:T ratios 5-9:1, depending of ILC3 yield per donor) (left). Representative histograms of RORγt expression by NK cells (control), ILC3s and ILC3s cultured with HuH6 cells (right). **(B)** Specific target cell lysis after 24h co-culture of indicated tumor targets with ILC3s (n=2-5 blood donors, E:T ratios 10-15:1). **(C)** NK cells were maintained in same conditions as ILC3s (NK cells^ILC3^) or with 400 U/ml IL-2 (NK cells) for 12h. Afterwards, cells were washed and co-cultured with HuH6 (up) or HepG2 cells (down) in the presence of 10 U/ml IL-2 for 24h. Specific target cell lysis was determined (n=3 blood donors, E:T ratios 9-12:1). ns, not significant, *p <0.05, by one-way Anova. **(D)** Sorted ILC3s were maintained in low (10 U/ml) or high (400 U/ml) concentration of IL-2 with or without IL-1β and IL-23 for 24h, prior to co-culture with HepG2 tumor targets (E:T ratios 5-12:1). Specific target cell lysis was determined for n=4 blood donors.

While NK cells are defined as cytolytic ILCs, able to directly kill tumor cells either *via* release of cytotoxic granules or through engagement of death-receptor ligands, ILC3s are considered to be cytokine producers (1, 29). So far, we observed that ILC3s can recognize and respond to tumor cells, resulting in increased IFN-γ production. Next, we investigated whether ILC3s could induce lysis of cancer cells, by measuring enzyme activity of lactate dehydrogenase (LDH) released by tumor cells over time in supernatants of ILC3-tumor cell co-cultures. Indeed, the presence of ILC3s increased lysis of HuH6 cells in a time-dependent manner (**Figure 2A**, left). Expression of RORγt by ILC3s remained stable during the co-culture (**Figure 2A**, right), indicating that ILC3s preserved their identity. Similar tumor cell lysis was observed in the co-culture of ILC3s and hepatocellular carcinoma (HepG2), while HuH7 (HCC cell line), cervical carcinoma (HeLa) and melanoma (SK-Mel-37, SK-Mel-28) cells were lysed to a lower extent (**Figure 2B**).

Although NK cells are considered prototypical cytolytic anti-tumor effectors, when exposed to IL-1β, IL-23 and IL-2, ILC3s induced significantly higher cancer cell lysis compared to NK cells (**Figure 2C**). In the absence of IL-1β and IL-23, and when concentration of IL-2 is increased for optimal NK cell activation (400 U/ml) (30, 31), NK cell cytotoxic activity was restored (**Figure 2C**). IL-2 (400 U/ml) might also favor ILC3 cytotoxic responses leading to increased tumor cell lysis (**Figure 2D**). These data indicate that ILC3s have the capacity to directly kill tumor targets, and that the microenvironmental condition might dictate the extent of their responses and the relative contribution of different effector cells to target cell lysis.

### ILC3 Cytotoxicity Is Mediated by Caspase-8

**Figure 3:**
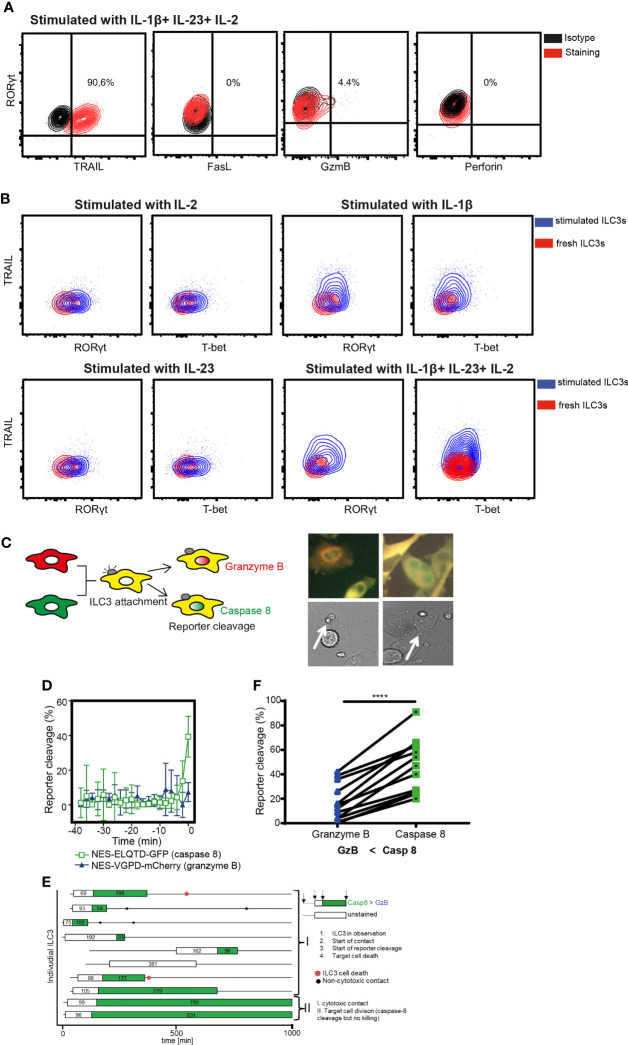
ILC3 cytotoxicity is mediated by Caspase-8. **(A)** Representative contour plots of TRAIL, FasL, Granzyme B and Perforin expression by ILC3s stimulated with indicated cytokines overnight. **(B)** Expression of TRAIL, RORγt and T-bet in freshly-isolated or in ILC3s stimulated with IL-1β, IL-23 and 10 U/ml IL-2 independently or combined. Data are representative of 2 different donors. **(C)** Cytokine-stimulated ILC3s were co-cultured with HeLa target cells expressing the NES-ELQTD-GFP-T2A-NES-VGPD-mCherry construct. Schematic representation of cytoplasmic and nuclear fluorescence signals in HeLa cells upon Granzyme B (GrzB) or Caspase-8 (Casp8) activation (left). Two examples of HeLa target cells with green nuclear signal upon interaction with ILC3 (white arrows), indicating Casp8 activity. **(D)** Four individual HeLa target cells were tracked over time and average reporter cleavage calculated upon contact with ILC3s (time point 0 set to time of cell death); mean ± SD. **(E)** Ten individual ILC3s were tracked over time. Each line represents one ILC3, every box visualizes ILC3-target cell contact, the length of the box displays the duration of the contact, and the color represents the reporter cleavage (green: Casp8 > GrzB). Non-cytotoxic contacts were indicated by black dots, and ILC3 cell death by a red star. **(F)** Quantified reporter cleavage at the time of maximum target cell death in 11 HeLa target cells upon contact with ILC3s (n=2 blood donors). ****p <0.0001, by paired Student’s t-test.

To dissect the mechanisms of ILC3-mediated the cytotoxicity, we first investigated the expression of death-receptor ligands and cytolytic effector molecules by ILC3s. Purified ILC3s exposed to IL-1β, IL-23, and IL-2 expressed TRAIL, but not FasL or Perforin (**Figure 3A** and **Supplementary Figure 2**). Granzyme B was detected only in a subset of cells (**Figure 3A**). Similar to RORγt, TRAIL expression was not detected in freshly-purified ILC3s (**Figure 3B**). TRAIL induction required the presence of IL-1β, while the amount of T-bet and RORγt could be increased when ILC3s were stimulated with IL-1β or IL-23 or IL-2 cytokines individually (**Figure 3B**). It was previously shown that the *TNFSF10* gene associated with H3K4Me2 modification in c-Kit+ ILCs from peripheral blood, which marks active and poised regulatory elements (27). At the same time, the authors did not detect *TNFSF10* among genes whose expression and poised epigenetic state overlaps, indicating that TRAIL-encoding transcript was also not detected in these cells. These data indicate transcriptional regulation of TRAIL expression by IL-1β.

To determine which pathway is used by ILC3s to lyse tumor cells, we applied a recently described method of time-lapse live-cell imaging (24). As tumor targets, we used the reporter HeLa cell line stably transduced with the NES-ELQTD-GFP-T2A-NES-VGPD-mCherry construct (26), leading to the equimolar expression of two fluorescent reporters: Granzyme B-sensitive nuclear export signal (NES) fused to mCherry and Caspase-8-sensitive NES fused to GFP. The NES retains both reporters in the cytosol, unless cleaved by Granzyme B or Caspase-8, which results in the diffusion of the fluorescent protein to the nucleus (**Figure 3C**). As such, Granzyme B and Caspase-8 activities can be assessed by measuring the corresponding fluorescence in the nucleus (24).

Measurements of the fluorescence signal in the nucleus of HeLa-reporter cells co-cultured with ILC3s, revealed an increased GFP compared to mCherry signal in the target cell nuclei at the event of cell death (**Figures 3D–F**), indicating mainly a Caspase-8-mediated apoptosis. Tumor cell apoptosis followed ILC3 and target cell interaction, indicating direct engagement of cell-cell contact between ILC3s and tumor cells (**Figures 3D, E** and **Supplementary Video 1**). Moreover, ILC3s did not appear to behave as serial killers, as during the indicated observation time, one ILC3 killed only one HeLa cell (**Figure 3E** and **Supplementary Video 1**). Taken together, these data demonstrate the ability of ILC3s to directly kill target cells *via* a Caspase-8-mediated mechanism.

### ILC3s Kill Tumor Cells *via* TRAIL-TRAILR2 Pathway

**Figure 4:**
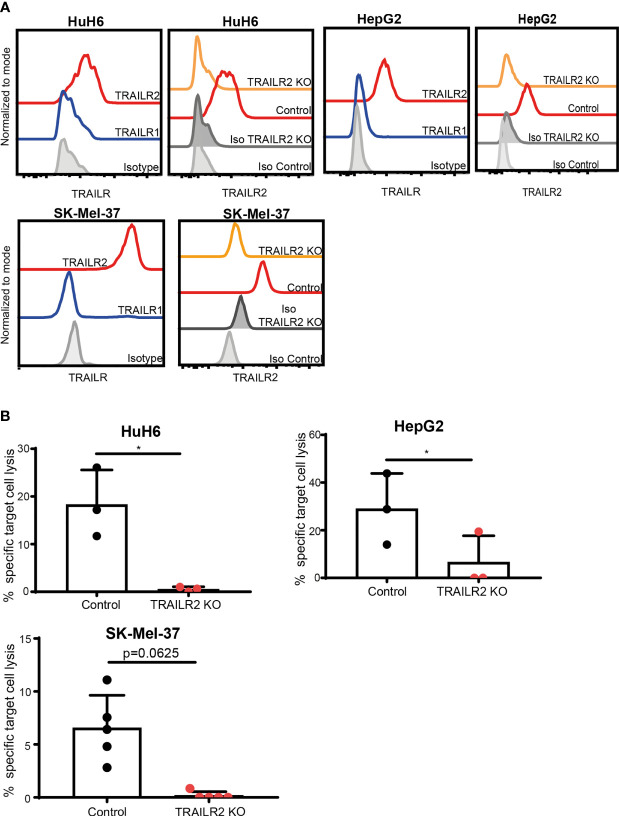
ILC3s kill tumor cells *via* TRAIL-TRAILR2 pathway. **(A)** Representative histograms of TRAILR1 and TRAILR2 expression by parental HuH6, HepG2 and SK-Mel-37 cells, and TRAILR2 expression by TRAILR2 KO and control cell lines expressing Cas9. **(B)** Specific target cell lysis of HuH6 TRAILR2 KO, HepG2 TRAILR2 KO, SK-Mel-37 TRAILR2 KO and the control cell lines expressing Cas9 after 24h co-culture with ILC3s (n=3 blood donors, E:T ratios 4-10:1). *p <0.05, by paired Student’s t-test (HepG2 and HuH6); p=0.0625 by Wilcoxon matched-pairs signed rank test (SK-Mel-37).

Since stimulated ILC3s expressed TRAIL, but not FasL, we next investigated whether TRAILR1 or TRAILR2 were expressed on the tumor cell lines HuH6, HepG2 and SK-Mel-37. While TRAILR1 was not detected, all three cell lines expressed TRAILR2 (**Figure 4A**). To investigate the role of TRAIL during the ILC3-mediated killing, we generated TRAILR2-deficient tumor cell lines using a CRISPR/Cas9-mediated gene deletion. All cells lines showed comparable apoptosis when exposed to TNF-α and cyclohexamide, indicating unaltered apoptotic machinery (**Supplementary Figure 3A**). Absence of TRAILR2 abrogated the lysis of tumor cells by ILC3s (**Figure 4B**). These results indicate that ILC3s use TRAIL-TRAILR2 receptor engagement to induce lysis of tumor cells. In support to these data, freshly-purified TRAIL^neg^ ILC3s were unable to induce tumor cell lysis to comparable levels as cytokine-activated ILC3s (**Supplementary Figure 3B**).

### Intra-Hepatic ILC3s Are Able to Kill Hepatic Tumor Cells

**Figure 5:**
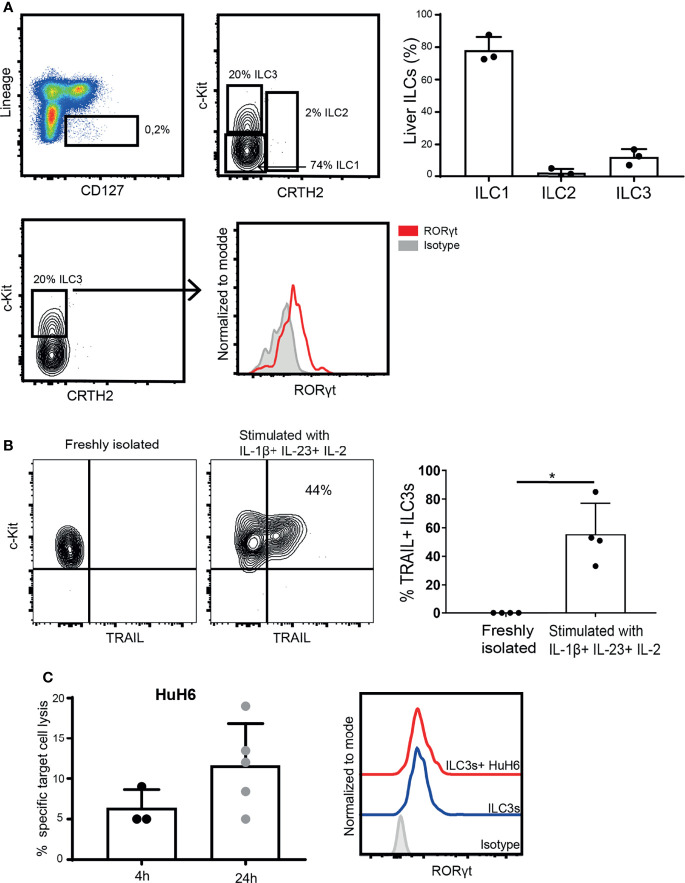
Intra-hepatic ILC3s are able to kill hepatic tumor cells. **(A)** Representative analysis of liver ILCs, gated as live CD45^+^, Lineage^-^ (CD3^-^, CD19^-^, CD14^-^, CD34^-^, CD94^-^, CD123^-^, TCR α/β^-^, TCR γ/δ^-^, FCϵR1α^-^), CD127^+^ cells. ILC1s were defined as c-Kit^-^ CRTH2^-^; ILC2s as c-Kit^+^ CRTH2^+^; and ILC3s as c-Kit^+^ CRTH2^-^ (left). Percentage of ILC1s, ILC2s and ILC3s among CD127^+^ Lin^-^ cells (n=3 liver samples from different donors, right). Representative histogram of RORγt expression by freshly-isolated intra-hepatic ILC3s (down). **(B)** Representative contour plot of c-Kit and TRAIL expression (left), and frequency of TRAIL-expressing ILC3s before and after exposure to cytokines (right, n=4 liver samples from different donors, *p <0.05 by Mann-Whitney test). **(C)** Specific target cell lysis of HuH6 cells after 4h and 24h of co-culture with cytokine-stimulated ILC3s (n=3-5 liver samples from different donors, E:T ratios 1-3:1, depending of ILC3 yield per donor). Representative histograms of RORγt expression by intra-hepatic ILC3s after the co-culture with HuH6 cells (right).

To determine if ILC3s can contribute to anti-tumor immune responses in tissues, we isolated ILC3s from liver tissue, and determined their lytic capacity. These tissue-resident ILC3s expressed RORγt (**Figure 5A**), but required stimulation with cytokines to upregulate TRAIL (**Figure 5B**). TRAIL-expressing ILC3s induced HuH6 target cell lysis, which increased over time (**Figure 5C**), while maintaining stable RORγt expression. These data indicate that, in the presence of type 3-stimulating cytokines, liver-resident ILC3s can participate in tumor cell lysis.

## Discussion

The function of tumor-infiltrating ILC3s, especially in primary human cancers is poorly explored. Although some studies reported the presence of ILC3s in tumor tissue (14–16), their role remains ambiguous. While described as tissue-protective, the ILC3 signature cytokine, IL-22, was shown to promote carcinogenesis, which depended on the balance between tissue regeneration and tumor development in the intestine (32). Conversely, the formation of tertiary lymphoid structures, supported by ILC3s, presented benefits for lung (14) and colorectal (23) cancer patients.

ILCs are considered to be tissue-resident and the first responders to tissue perturbation. While local alarmins can provoke early responses of resident ILCs, the inflammatory reaction can lead to an increase in ILC numbers in inflamed tissue. Currently, the relative contribution of local proliferation versus differentiation from tissue precursors to the increased ILC numbers during an immune reaction remains unclear. Moreover, it was also shown that certain ILC subsets could circulate towards sites of inflammation to support ongoing immune responses (33). Recently, two publications suggested that human blood ILCs provided a pool of precursor cells, which can be recruited to the tissues and develop into mature ILCs under local environmental cues (27, 28). Lim et al. showed that c-Kit+ CRTH2- ILCs from the blood did not express RORγt, unless exposed to IL-23 and IL-1β (27, 28), which is in concordance with our observations. Furthermore, ILC2s and ILC3s could be effectively recruited to the type 1 responses, by acquiring the ability to produce IFN-γ through cellular plasticity, including upregulation of transcription factor T-bet (3, 34). Cytokines, such as IL-12 and/or IL-15 could re-shape ILC functions, broadening the spectrum of their functional responses (3, 35, 36). Indeed, in the presence of IL-12, NKp46+ ILC3s suppressed tumor growth of mouse melanoma (18). Recently, it was shown that IL-12 could support the acquisition of ILC3 cytotoxicity by tonsillar ILC3s by inducing the expression of cytolytic molecules, along with several NK-expressed molecules, including CD94 and Eomes (22). Of note, these ILC3s preserved expression of RORγt. Similarly, CD127+CD94+ ILCs identified in human *lamina propria* were reported to secrete granulysin in response to IL-15 and to accumulate in Crohn’s disease patients (37). The ability to eliminate K562 cells or to degranulate upon their encounter was also demonstrated earlier for human tonsillar and intestinal intraepithelial ILCs (38), NKp44+ ILC3s exposed to IL-12 and IL-15 *in vitro* (36), and for tonsillar CD94+ ILCs, generated from non-cytotoxic fraction when exposed to IL-12 (22). In these studies, ILCs were derived from tissues, such as pediatric tonsils or intestine, where their phenotype was pre-shaped by local microenvironments, and frequently further expanded using feeder cells and cytokines. In contrast, peripheral blood Lin-CD127+c-Kit+ cells, that by phenotype correspond to tissue ILC3s, were shown to rather display “naive” progenitor-like features and were able to generate other ILC lineages *in vitro* (27). Accordingly, these cells neither expressed ILC lineage-determining transcription factors, such as RORγt, GATA-3 or Eomes, receptors NKp44, NKp80, CD16 or CD94, nor were able to secrete IL-17 and IL-22, in contrast to their gut-derived counterparts (27). In addition, NKp44-(CD62L-) tonsillar ILC3 subset was also suggested to represent a naive ILC3 state and did not produce IL-17 and IL-22 in response to IL-23 and IL-1β (39). Here, we describe that peripheral blood-derived ILC3-like cells can acquire RORγt and TRAIL expression upon short 16-24h exposure to type 3 cytokines. In contrast to previously described CD94+ cytotoxic ILCs, these cells rather exerted perforin-independent, TRAIL-mediated killing of tumor targets. IL-1β was necessary to induce expression of TRAIL, while NK cell-like features (Eomes, NKp80, CD16, and CD94) were not detected. In addition, ILC3s can produce TNF-α, which might also contribute to Caspase-8 activation in susceptible target cells (40). These data together indicate that ILC3 functional specialization might be even broader than previously anticipated, allowing these cell population to acquire different modes of cytolytic responses (perforin vs TRAIL) driven by different environmental conditions (type 1 vs type 3 cytokines).

Type 1 immune responses are considered beneficial for effective anti-tumor immunity, and NK cells and T cells (CD8+ cytotoxic T lymphocytes and CD4+ Th1 cells) are considered prototypical producers of IFN-γ. Recently it was shown that blood- and tonsil-derived ILC3s were also able to produce IFN-γ in response to IL-23 and IL-1β (10). Here, we show that ILC3 IFN-γ production can be further increased upon interaction with tumor cells. Therefore, IFN-γ+ ILC3s might contribute to a pro-inflammatory environment that promotes efficient anti-tumor responses. It remains to be determined which recognition mechanisms and signaling machineries contribute to tumor-induced IFN-γ release by ILC3s. It was previously shown that subset(s) of ILC3s can express certain NK cell receptors, including NKp44 and NKp30 (11), whose ligands are frequently upregulated by malignant cells. Moreover, ILC3s could also be regulated by soluble factors, including both cytokines and metabolites that can be released by tumor cells. In the tumor microenvironment, these factors could additively contribute to ILC3 functions.

In addition to IFN-γ release upon tumor cell encounter, here, we show that both blood- and tissue-derived ILC3s were able to directly lyse tumor cells. This function was exerted under type 3 conditions, where ILC3s maintained RORγt expression, therefore excluding their differentiation towards an ILC1-like phenotype (1). The transcription factor T-bet that is detected in freshly-isolated ILC3s and upregulated upon their exposure to cytokines, might contribute to the ability of these cells to secrete IFN-γ. In this microenvironment, NK cells were unable to induce comparable target cell lysis. This might be of high relevance for the transformed tissues with negligible type 1 immunity. Accordingly, it was recently shown that IL-23 was overexpressed in human HCCs (41) and melanoma (42), as well as in other tumor tissues, such as colorectal, ovarian and bladder cancer (43). It could be speculated that ILC3s might provide an alternative source of not only IFN-γ, but also cytotoxicity, in the tumors with a type 3 microenvironment, and their functions might even increase with higher availability of IL-2. This can also be relevant for early tumor development in tissues enriched with local resident ILC3s, such as gut, where they can provide early elimination of transformed cells, before type 1 immunity emerges.

We showed that ILC3s preferentially used TRAIL and TRAILR2 engagement to kill their targets. In certain tissues, both developing tumors and arriving metastasizing disseminated cells were shown to be TRAIL-sensitive, and TRAIL represents important pathway for their elimination and control. These includes liver cancer (44), melanoma (45) and breast cancer (46). In these tissues, NK cells were considered main anti-tumor cytotoxic effectors. In addition, some tissues, such as liver, are enriched in ILC1s, which also can exert TRAIL-mediated cytotoxicity, although the proportion of TRAIL-expressing ILC1s in human liver might be lower compared to mouse (47). Due to their residency, ILC1s and ILC3s might be able to provide protection even before NK cells are recruited. Which cells are preferentially engaged might be dictated by the microenvironment, and might dynamically change in a spatiotemporal manner as the response progresses. Although we also detected Granzyme B expression in a subset of ILC3s, these ILC3s did not express perforin. These data suggest that Granzyme B might have other functions, alternative to inducing target cell apoptosis. It was recently shown that ILC3s from the gut were able to express Granzyme B upon bacterial stimulation (48). Furthermore, Granzyme B has the ability to remodel the extracellular matrix by inducing anoikis and detachment of tumor cells, which prevents tumor cell migration and invasion (49). Therefore, Granzyme B produced by ILC3s might play a perforin-independent anti-metastatic function.

Taken together, our results revealed novel insights into the role of ILC3s in anti-tumor immunity. Our findings demonstrate that ILC3s can display anti-tumor functions, including the release of pro-inflammatory cytokines, and direct lysis of tumor targets *via* TRAIL-mediated cytotoxicity. Thus, we suggest that ILC3s could act as first-line local responders in tissues against transformed or metastasizing cells, which might be further exploited for therapies against cancer.

## Data Availability Statement

The original contributions presented in the study are included in the article/**Supplementary Material**. Further inquiries can be directed to the corresponding authors.

## Ethics Statement

The studies involving human participants were reviewed and approved by Ethik Kommission II of the Medical Faculty Mannheim. The patients/participants provided their written informed consent to participate in this study.

## Author Contributions

J-JS designed and performed experiments, analysed data and wrote the manuscript. TH and IP performed experiments and provided technical help. EB, NR, and CW provided material and helpful discussions. AS performed experiments, provided helpful discussions, wrote and revised the manuscript. MC and AC supervised the project, designed experiments and revised the manuscript. All authors contributed to the article and approved the submitted version.

## Funding

The project was supported by grants from the German Research Foundation: SFB1366 (Project number 394046768-SFB 1366; C02 to AC), SPP 1937 (CE 140/2-2 to AS and AC), CEECINST/00091/2018 (to MC), TRR179 (TP07 to AC), SFB-TRR156 (B10N to AC), RTG 2099 (Project number: 259332240 - RTG2099; P9 to AC), a network grant of the European Commission (H2020-MSCA-MC-ITN-765104-MATURE-NK), and by the DKFZ-MOST (CA172).

## Conflict of Interest

The authors declare that the research was conducted in the absence of any commercial or financial relationships that could be construed as a potential conflict of interest.

## Publisher’s Note

All claims expressed in this article are solely those of the authors and do not necessarily represent those of their affiliated organizations, or those of the publisher, the editors and the reviewers. Any product that may be evaluated in this article, or claim that may be made by its manufacturer, is not guaranteed or endorsed by the publisher.

## Supplementary Material

Supplementary Figure 1
**(A)** Representative analysis of 2B4 expression on freshly-isolated blood ILC3s [gated as live, CD45^+^, Lineage^-^ (CD3^-^, CD19^-^, CD14^-^, CD34^-^, CD94^-^, CD123^-^, TCR α/β^-^, TCR γ/δ^-^, FCϵR1α^-^), CD127^+^, c-Kit^+^ and CRTH2^-^ cells], NK cells [gated as live, CD45^+^, Lineage^-^ (CD3^-^, CD19^-^, CD14^-^, CD34^-^, CD123^-^, TCR α/β^-^, TCR γ/δ^-^, FCϵR1α^-^), CD127^-^, CD56^+^ and NKp80^+^ cells], and ILC3s upon 24h stimulation with 100 ng/ml IL-1β, 100 ng/ml IL-23 and 10 U/ml IL-2. Data for n=2 blood donors are shown. **(B)** Histograms (left) depict representative staining of T-bet on NK cells and ILC3s treated as in A (FMO, control staining without antibody). Quantification of T-bet expression by ILC3s normalized to isotype control (n=4 blood donors, *** p<0.001 by unpaired Student’s t-test).

Supplementary Figure 2
**(A)** Representative analysis of Perforin expression in freshly-isolated blood NK cells (gated as live, CD45^+^,CD3^-^, CD19^-^, CD14^-^, CD56^+^), and in blood ILC3s (purified as live, CD45^+^, Lineage^-^ (CD3^-^, CD19^-^, CD14^-^, CD34^-^, CD94^-^, CD123^-^, TCR α/β^-^, TCR γ/δ^-^, FCϵR1α^-^), CD127^+^, c-Kit^+^ and CRTH2^-^ cells) stimulated 20h with 100 ng/ml IL-1β, 100 ng/ml IL-23 and 10 U/ml IL-2. As control for specific staining, cells were treated with corresponding isotype control. Data are representative of 2 donors. **(B)** Primary human NK cells were cultured 2 days in IL-2-supplemented media (400 U/ml) and then stimulated with IL-2 alone or together with 100 ng/ml of IL-18 and 10 ng/ml of IL-12 for 12 hours. FasL expression was analyzed by flow cytometry.

Supplementary Figure 3
**(A)** Control (WT) and TRAILR2 KO cells were seeded 24h before treatment with 20 ng/ml of TNF-α and 50 μg/ml of cycloheximide. After 6h, cells were collected and stained with Annexin V. **(B)** Blood ILC3s isolated as live, CD45^+^, Lineage^-^ (CD3^-^, CD19^-^, CD14^-^, CD34^-^, CD94^-^, CD123^-^, TCR α/β^-^, TCR γ/δ^-^, FCϵR1α^-^), CD127^+^, c-Kit^+^ and CRTH2^-^ cells, freshly-purified or stimulated with 100 ng/ml IL-1β, 100 ng/ml IL-23 and 10 U/ml IL-2 for 24h, were co-cultured 24h with HepG2 tumor targets. Target cell lysis was determined by measurement of specific LDH release in the cell culture supernatants. Data are visualized using R 4.1.0 and ggplot2, linear prediction curve is depicted (left). Bar graph shows summarized data for n=4-7 donors and E/T ratios of 5-25:1. *p<0.05 by Welch’s t test.

Supplementary Video 1 16h time-lapse video of ILC3 and HeLa cell co-culture. 350-qm-wide micro well contains HeLa-CD48 target cells (large and adherent) stably expressing the NES-ELQTD-GFP-T2A-NES-VGPD-mCherry construct and blood-derived ILC3s (small round shaped). Example of reporter cleavage and cell death upon ILC3-target cell contact is depicted at 0:22 min. The green fluorescence in the nucleus indicates Caspase-8 activity.
