# Supplementary figures and images for: Human ILC3 Exert TRAIL-Mediated Cytotoxicity Towards Cancer Cells
Source: Front Immunol. 2022 Mar 1;13:742571. doi: 10.3389/fimmu.2022.742571 (PMC8921484; doi:10.3389/fimmu.2022.742571)

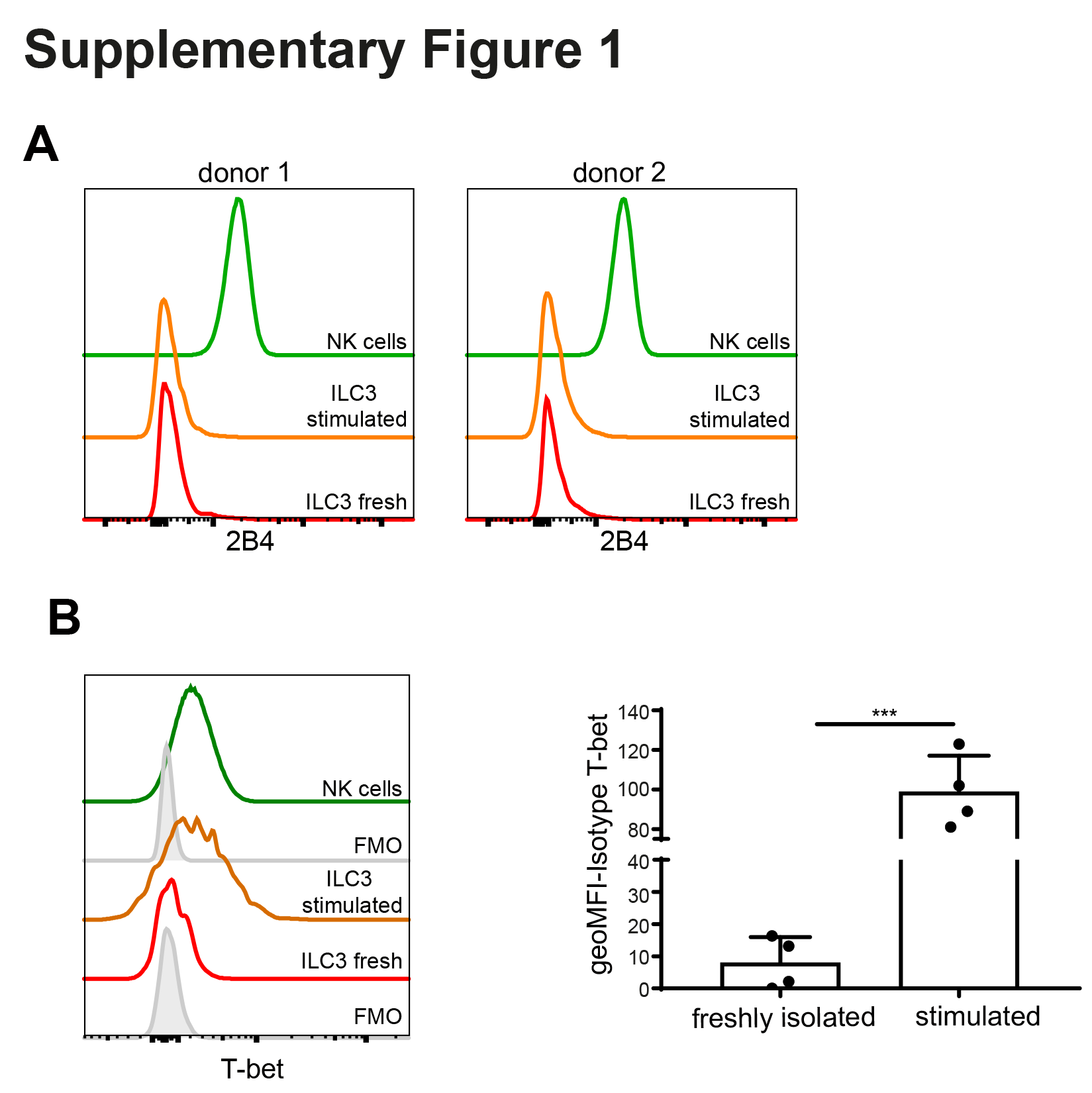

Supplement: Supplementary Figure 1 — (A) Representative analysis of 2B4 expression on freshly-isolated blood ILC3s [gated as live, CD45+, Lineage- (CD3-, CD19-, CD14-, CD34-, CD94-, CD123-, TCR α/β-, TCR γ/δ-, FCϵR1α-), CD127+, c-Kit+ and CRTH2- cells], NK cells [gated as live, CD45+, Lineage- (CD3-, CD19-, CD14-, CD34-, CD123-, TCR α/β-, TCR γ/δ-, FCϵR1α-), CD127-, CD56+ and NKp80+ cells], and ILC3s upon 24h stimulation with 100 ng/ml IL-1β, 100 ng/ml IL-23 and 10 U/ml IL-2. Data for n=2 blood donors are shown. (B) Histograms (left) depict representative staining of T-bet on NK cells and ILC3s treated as in A (FMO, control staining without antibody). Quantification of T-bet expression by ILC3s normalized to isotype control (n=4 blood donors, *** p<0.001 by unpaired Student’s t-test). [file Image_1.tif]

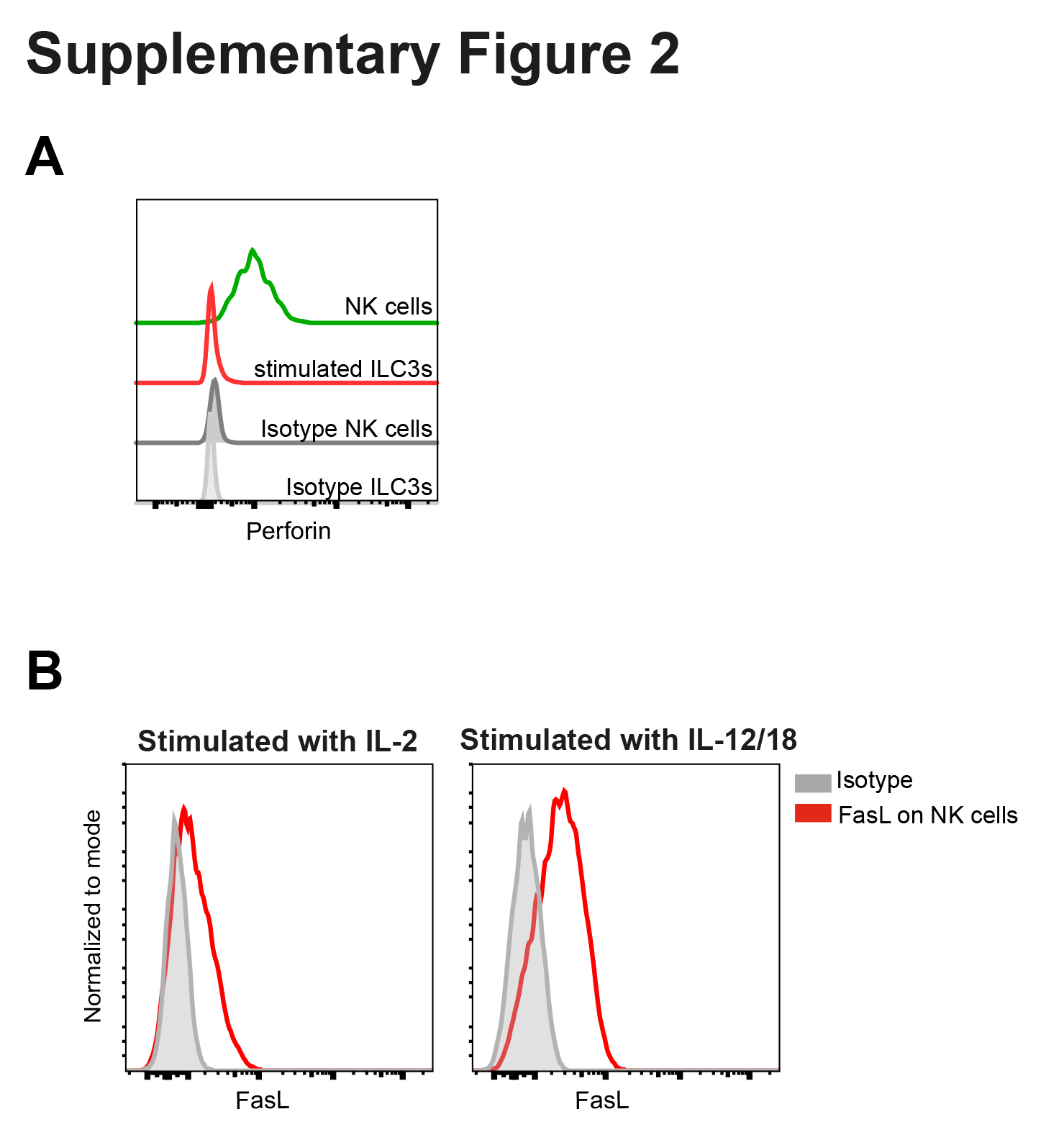

Supplement: Supplementary Figure 2 — (A) Representative analysis of Perforin expression in freshly-isolated blood NK cells (gated as live, CD45+,CD3-, CD19-, CD14-, CD56+), and in blood ILC3s (purified as live, CD45+, Lineage- (CD3-, CD19-, CD14-, CD34-, CD94-, CD123-, TCR α/β-, TCR γ/δ-, FCϵR1α-), CD127+, c-Kit+ and CRTH2- cells) stimulated 20h with 100 ng/ml IL-1β, 100 ng/ml IL-23 and 10 U/ml IL-2. As control for specific staining, cells were treated with corresponding isotype control. Data are representative of 2 donors. (B) Primary human NK cells were cultured 2 days in IL-2-supplemented media (400 U/ml) and then stimulated with IL-2 alone or together with 100 ng/ml of IL-18 and 10 ng/ml of IL-12 for 12 hours. FasL expression was analyzed by flow cytometry. [file Image_2.tif]

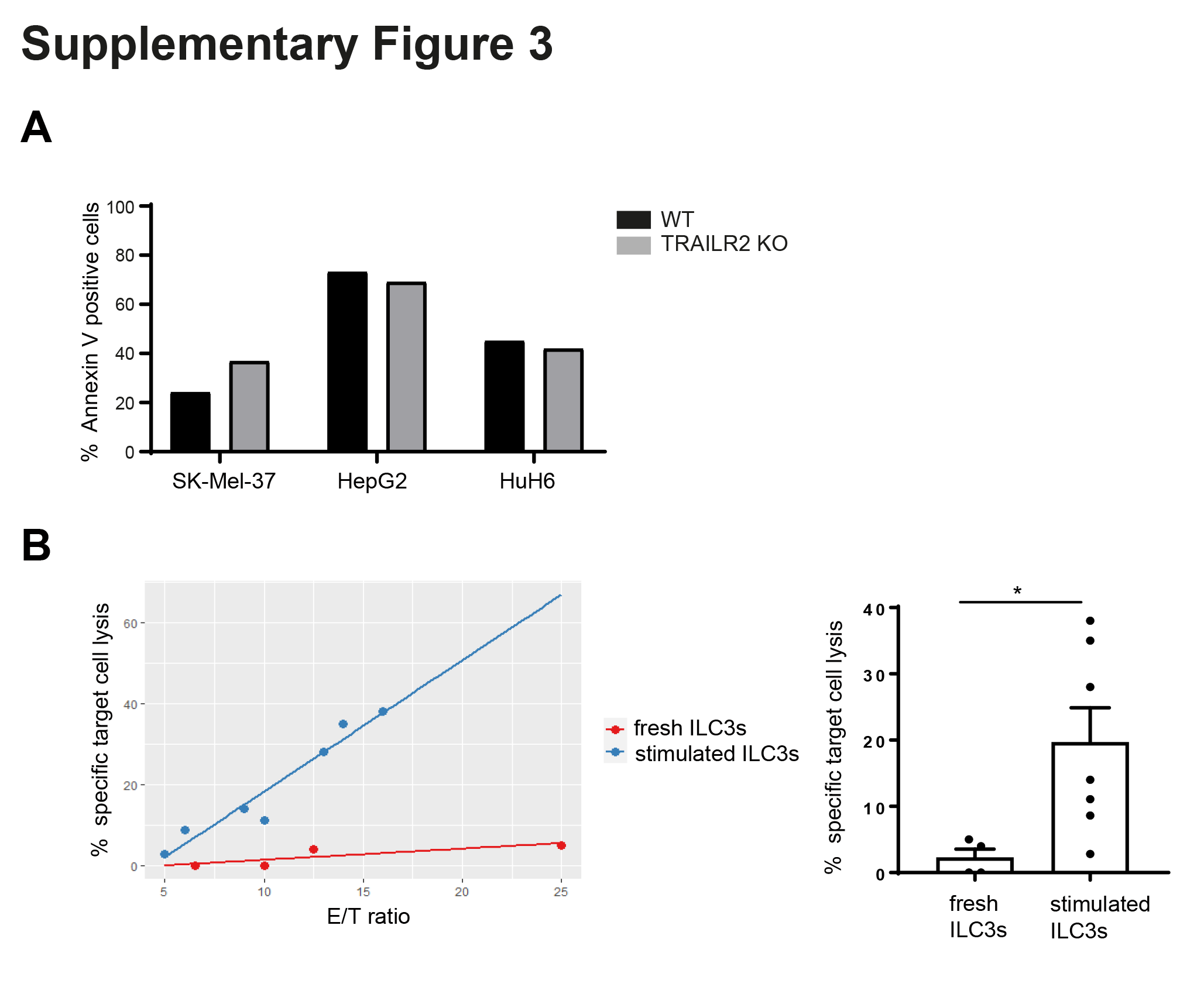

Supplement: Supplementary Figure 3 — (A) Control (WT) and TRAILR2 KO cells were seeded 24h before treatment with 20 ng/ml of TNF-α and 50 μg/ml of cycloheximide. After 6h, cells were collected and stained with Annexin V. (B) Blood ILC3s isolated as live, CD45+, Lineage- (CD3-, CD19-, CD14-, CD34-, CD94-, CD123-, TCR α/β-, TCR γ/δ-, FCϵR1α-), CD127+, c-Kit+ and CRTH2- cells, freshly-purified or stimulated with 100 ng/ml IL-1β, 100 ng/ml IL-23 and 10 U/ml IL-2 for 24h, were co-cultured 24h with HepG2 tumor targets. Target cell lysis was determined by measurement of specific LDH release in the cell culture supernatants. Data are visualized using R 4.1.0 and ggplot2, linear prediction curve is depicted (left). Bar graph shows summarized data for n=4-7 donors and E/T ratios of 5-25:1. *p<0.05 by Welch’s t test. [file Image_3.tif]
